# Deubiquitination Reactions on the Proteasome for Proteasome Versatility

**DOI:** 10.3390/ijms21155312

**Published:** 2020-07-27

**Authors:** Ji Yeong Shin, Srinivasan Muniyappan, Non-Nuoc Tran, Hyeonjeong Park, Sung Bae Lee, Byung-Hoon Lee

**Affiliations:** 1 Department of New Biology, Daegu Gyeongbuk Institute of Science and Technology (DGIST), Daegu 42988, Korea; shinjy@dgist.ac.kr (J.Y.S.); srinivasan@dgist.ac.kr (S.M.); tnnuoc_88@dgist.ac.kr (N.-N.T.); parkhj0829@dgist.ac.kr (H.P.); 2 Protein Dynamics-based Proteotoxicity Control Lab, Basic Research Lab, Daegu Gyeongbuk Institute of Science and Technology (DGIST), Daegu 42988, Korea; sblee@dgist.ac.kr; 3 Center for Cell Fate Reprogramming & Control, Daegu Gyeongbuk Institute of Science and Technology (DGIST), Daegu 42988, Korea; 4 Department of Brain & Cognitive Sciences, Daegu Gyeongbuk Institute of Science and Technology (DGIST), Daegu 42988, Korea

**Keywords:** proteasome, proteolysis, ubiquitin, deubiquitination, deubiquitinating enzyme, USP14, UCH37, RPN11

## Abstract

The 26S proteasome, a master player in proteolysis, is the most complex and meticulously contextured protease in eukaryotic cells. While capable of hosting thousands of discrete substrates due to the selective recognition of ubiquitin tags, this protease complex is also dynamically checked through diverse regulatory mechanisms. The proteasome’s versatility ensures precise control over active proteolysis, yet prevents runaway or futile degradation of many essential cellular proteins. Among the multi-layered processes regulating the proteasome’s proteolysis, deubiquitination reactions are prominent because they not only recycle ubiquitins, but also impose a critical checkpoint for substrate degradation on the proteasome. Of note, three distinct classes of deubiquitinating enzymes—USP14, RPN11, and UCH37—are associated with the 19S subunits of the human proteasome. Recent biochemical and structural studies suggest that these enzymes exert dynamic influence over proteasome output with limited redundancy, and at times act in opposition. Such distinct activities occur spatially on the proteasome, temporally through substrate processing, and differentially for ubiquitin topology. Therefore, deubiquitinating enzymes on the proteasome may fine-tune the degradation depending on various cellular contexts and for dynamic proteolysis outcomes. Given that the proteasome is among the most important drug targets, the biology of proteasome-associated deubiquitination should be further elucidated for its potential targeting in human diseases.

## 1. Introduction

In eukaryotes, the 26S proteasome is a multi-subunit protease complex primarily responsible for the degradation of intracellular proteins which have been marked by ubiquitin tags [1]. This allows proteostasis—the equilibrium and optimal biological function of cellular proteins—to be maintained [2]. The proteasome holoenzyme consists of the 20S core particle (CP) and one or two 19S regulatory particles (RPs). The cylindrical CP is a hollow, four stacked heteroheptameric ring structure (α_1-7_β_1-7_β_1-7_α_1-7_) containing three types of proteolytic active sites embedded in each central β-ring [3]. Its outer α-rings serve as a gated channel for substrate entry [4]. The 19S RP mediates a series of enzymatic and non-enzymatic multi-step processes that are required for selective degradation of ubiquitinated conjugates, including ubiquitin-specific substrate recognition, disassembly of ubiquitin chains, and mechanical unfolding and translocation of the substrate into the inner CP [3,5].

Considering the strict requirement of ubiquitin modification for proteasomal recognition of substrates, it is striking that the human proteasome contains three distinct classes of deubiquitinating enzymes (DUBs)—two cysteine proteases, USP14 (Ubp6 in yeast) and UCH37/UCH-L5, and the metalloprotease RPN11/PSMD14/POH1 [1]. USP14 activity is reversibly associated with the proteasome, and shows extraordinary multi-chain specific cleavage of ubiquitin conjugates [6,7]. USP14-mediated deubiquitination is also competent for rescuing substrates prior to the irreversible commitment of proteasomal proteolysis [6,8]. In contrast, RPN11 is an integral subunit of the proteasome, and displays ATP-stimulated deubiquitination directly coupled to substrate degradation [9,10]. Interestingly, both RPN11 and USP14 show apparent en bloc cleavage in which whole ubiquitin chains are released by proximal cutting [6,11]. This indicates that, in spite of having similar cleavage mechanisms, deubiquitination reactions on the proteasome may lead to different proteolytic outcomes. The exact function of UCH37 is less clear, but this enzyme is speculated to edit the ubiquitin chains, yielding repressed or enhanced degradation of substrates depending on the proteasomal demand [12,13,14].

Recent cryo-electron microscopy (cryo-EM) studies have revealed a multitude of conformational states of the proteasome both in substrate-free and substrate-bound forms [5,15]. These conformational dynamics provide the structural basis for the speculation that degradation kinetics can occur progressively from initial substrate engagement to irreversible commitment, and finally to proteolytic cleavage [16]. Thus, for proteasomal versatility, it is likely that proteasome-bound DUBs modulate degradation events by differentially or even co-operatively handling ubiquitin polymers in accordance with substrate processing steps. A growing body of evidence also suggests that, in addition to their apparent catalytic effects, DUBs act non-catalytically on the proteasome by allosterically influencing its conformational states, an observation which points to their multi-faceted complexity [17,18,19]. In this article, we concisely summarize the current understanding of proteasome-associated DUBs, focusing on their mechanisms of action and roles in cell physiology. Recently-developed inhibitors, which specifically target proteasomal DUBs, will also be briefly discussed, since they not only serve as dissecting tools but also have therapeutic potential in human disease [20].

## 2. A Structural View of Proteasomal Deubiquitinating Enzymes

### 2.1. Ubp6/USP14

**Figure 1:**
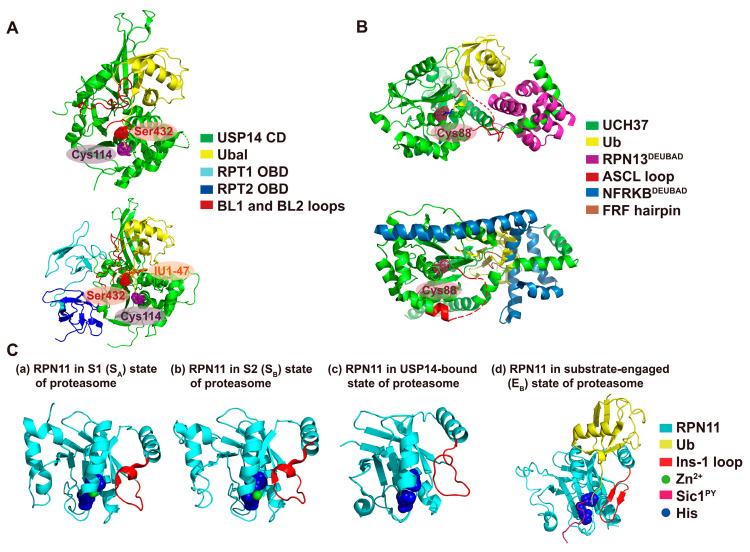
Structures and conformational changes of proteasomal DUBs. (**A**) (Top panel) The crystal structure of the USP14 catalytic domain (CD) as a free form (PDB: 2AYN) [24]. Ubiquitin aldehyde (Ubal) is positioned on USP14’s catalytic domain as its inactive state to show steric hindrance of ubiquitin with BL1 and BL2 loops. The active site residues of USP14, including the catalytic Cys114 and conserved Ser432, are colored as stippled violet and red spheres, respectively. (Bottom panel) The structure of USP14-Ubal in human 26S proteasome complex that is resolved by cryo-EM (PDB: 5GJQ) [26]. Only the interactions between USP14 and OB domains (OBDs) of RPT1 and RPT2 are shown. IU1-47 was positioned by superimposing the crystal structure of IU1-47-bound USP14 (PDB: 6IIL) [28]. IU1-47 is shown as an orange stick model. (**B**) (Top panel) Structure of UCH37 in complex with RPN13^DEUBAD^ domain and ubiquitin (PDB: 4UEL) [29]. The catalytic Cys88 of UCH37 is colored as stippled raspberry spheres. The ASCL loop is not fully resolved in the crystal structure, so the unresolved portion of the loop is indicated as a dashed red line. (Bottom panel) Structure of UCH37 in complex with NFRKB^DEUBAD^ domain (PDB:4UF5) [29]. Ubiquitin is modeled on this complex based on the structure of UCH37-RPN13^DEUBAD^-ubiquitin and shows the steric hindrance with NFRKB^DEUBAD^. (**C**) Structure and conformational changes of RPN11 in the context of dynamic conformational states of the proteasome. Panels (a)–(d) represent each conformation of RPN11 in S1 (PDB: 5T0G) [30], S2 (PDB: 5T0H) [30], USP14-Ubal-bound human proteasome (PDB: 5GJQ) [26] and substrate-engaged human 26S proteasome (PDB: 6MSE) [31], respectively. RPN11’s active-site histidines are shown as a blue sphere model. Color coding of proteins and regions is given in the key.

Roughly 11–40% of isolated proteasomes are transiently associated with Ubp6/USP14, suggesting the dynamic influence of this DUB over proteolysis [19,21,22]. Among the most striking features of Ubp6/USP14 is its 300–800 fold activation upon binding to the proteasome [8,23]. This isopeptidase activity rapidly deubiquitinates multi-chain ubiquitin conjugates en bloc, and thus can counteract substrate degradation by attenuating its affinity for the proteasome [6,8]. In contrast, the free form of USP14 exhibits only sluggish deubiquitinating activity, as the close examination of its structure revealed that USP14’s active site is occluded by two crossover loops (BL1 and BL2), which block the access of the C-terminus of ubiquitin (Figure 1A, top panel) [24]. Thus, de-repression of the autoinhibitory state of USP14 must be achieved by its interaction with the proteasome. The N-terminal ubiquitin-like (UBL) domain of Ubp6/USP14 is the primary binding region for the proteasome, which tethers to the T2 site of RPN1 [8,23,25]. Recent cryo-EM studies of Ubp6/USP14-bound proteasomes further elucidated that the catalytic domain is also bound to the ATPase ring through the OB domains of RPT1 and RPT2; this binding shifts the conformation of proteasome from the S1 (resting) to the S2 (intermediate) state [21,26]. The interaction displaces the BL1 and BL2 loops from USP14’s active site and allows the ubiquitin C-terminus free access to the catalytic cysteine [26] (Figure 1A, bottom panel). Intriguingly, USP14 can be also activated in a proteasome-independent manner by AKT-mediated phosphorylation of the Ser432 residue [27]. This highly conserved Ser432 is located within the BL2 loop, in close proximity to a negatively charged patch on the thumb region of USP14 (Figure 1A). Phosphorylation of Ser432 may push the BL2 loop away from the thumb region and relieve its inhibitory effect on USP14 [13].

Apart from activation by proteasome, Ubp6/USP14 can, in turn, allosterically regulate the proteasome activity; this enzyme induces CP gate opening, stimulates the ATPase activity of RP, and inhibits substrate degradation through a non-catalytic mechanism [17,18,19,32,33] (see Section 3 for more discussion). The non-catalytic effect of Ubp6/USP14 may suggest that this DUB stalls the proteasome in the S2 state and prevents its conformational transition back to the substrate-free S1 state and a productive round of the degradation cycle [3,13,18].

Emerging evidence has clearly demonstrated that specific inhibition of USP14 by small-molecules accelerates the degradation of a subset of substrates by enhancing proteasomal activity [8,13,34,35]. Recent co-crystal structures of USP14 with small-molecule USP14 inhibitors—IU1, IU1-47, and other derivatives—have shown that these compounds bind to the thumb-palm cleft pocket near the USP14 catalytic center (Figure 1A, bottom panel) [28,36]. This unique positioning occludes the access of the C-terminus of ubiquitin to the active site Cys114 only when the two blocking loops are displaced, providing the mechanistic basis for their selective inhibition of proteasome-associated USP14, but not the free form [3].

### 2.2. UCH37

Proteasome-bound UCH37 is known to exhibit poor DUB activity for isopeptide-linked ubiquitin–protein conjugates, despite showing predominant activity over USP14 against ubiquitin adducts with small leaving groups, such as ubiquitin amidomethylcoumarin (UbAMC) [8,37]. Similar to USP14, UCH37’s DUB activity can be also markedly increased upon binding to RPN13, a ubiquitin receptor on the proteasome [14,38]. Autoinhibition of the apo form of UCH37 is mainly due to (1) the inhibitory position of UCH37-like (ULD, aka RPN13-binding) domain onto the catalytic domain and (2) the active site crossover loop (ASCL), a flexible segment placed just over the catalytic cysteine (Figure 1B). This topology may restrict the cleavage of isopeptide-linked ubiquitin conjugates and allow only moderate active site access to ubiquitin with a small leaving group. Upon binding to the deubiquitinase adaptor (DEUBAD) domain of RPN13, the ULD domain is redirected to relieve its autoinhibitory effect on UCH37, and also the ASCL loop is modestly displaced from its autoinhibitory position for ubiquitin binding with less hindrance (Figure 1B, top panel) [29,39]. A unique feature of UCH37 is that this enzyme also serves as a member of the chromatin-remodeling complex INO80 [40]. The UCH37-binding subunit within the INO80 complex is NFRKB and binding also occurs through its DEUBAD domain. However, unlike RPN13, the NFRKB^DEUBAD^ domain represses UCH37’s DUB activity, primarily because the FRF hairpin in NFRKB^DEUBAD^ blocks the ubiquitin docking site directly [29,39]. In addition, its C-terminal long helix makes the inhibitory contact against the catalytic domain of UCH37 while stabilizing the ULD domain into an inhibitory position (Figure 1B, bottom panel) [29,39].

**Figure 2:**
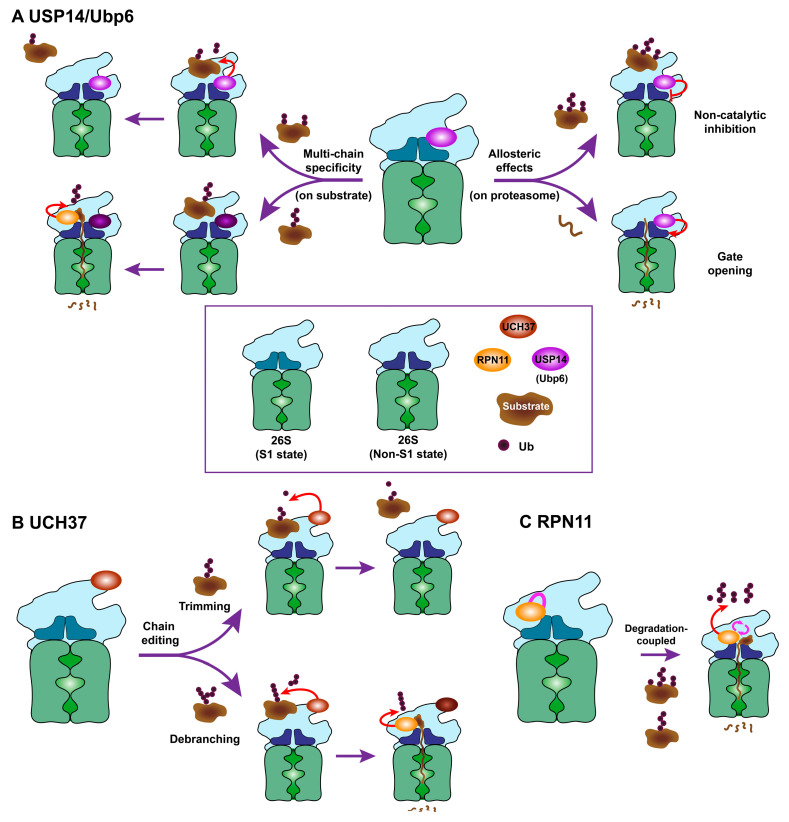
Proposed working models for proteasomal DUBs. (**A**) (Left panel) Multi-chain specific and en bloc cleavage mechanism by USP14/Ubp6-mediated deubiquitinating activity. As shown in the upper panel, USP14/Ubp6 cleaves multi-chain ubiquitin conjugates at the proximal site. Depending on the characteristics of the remaining chain, the substrate deubiquitinated by USP14/Ubp6 can be spared from degradation prior to the commitment step. The lower panel indicates that a single chain bearing ubiquitin–protein conjugate serves as a poor USP14/Ubp6 substrate. This conjugate still can be subject to RPN11 activity, and subsequently undergoes degradation by the proteasome. (Right panel) USP14/Ubp6’s allosteric regulation on the proteasome. The upper panel shows that USP14/Ubp6 negatively regulates proteasome activity through its non-catalytic mechanism. In the lower panel, the model indicates that binding of USP14/Ubp6 to the proteasome in a certain conformational state (i.e., S2 state) induces the gate opening of the CP and enhances the uptake or degradation of the peptide substrate or some unstructured proteins. (**B**) Putative chain editing mechanism of UCH37 on the proteasome. In the upper panel, UCH37 acts by progressively trimming each mono-ubiquitin from the distal end of chains. This distal trimming may decrease the dwell time of substrates on the proteasome and thus rescue the substrate from degradation. The lower panel shows a recently reported debranching activity of UCH37 [14]. In contrast to the upper model, K48-linked branched chains can be selectively debranched by UCH37, resulting in promoted degradation by proteasome. (**C**) A typical working mechanism of degradation-coupled deubiquitination by RPN11. As ubiquitinated substrates are engaged and committed to degradation, RPN11 is shifted close to the substrate entry port of the ATPase ring. The Ins-1 loop of the active site also undergoes its conformational switch into a catalytically permissive β-hairpin structure, as depicted. Thus, RPN11 on the proteasome forms a functional module of deubiquitination activity, which is coupled to the unfolding–translocation–degradation axis. Where appropriate, DUB ovals that are darkly-colored inside indicate the inactive state of each enzyme.

The substrate specificity of UCH37 remains elusive (see also Section 4), but recent structural evidence implies that its physiological substrates must be placed as the folded domain being on the same side of the ASCL as the bound ubiquitin [13,39]. This seemingly strict requirement for favored UCH37 substrates may explain why UCH37 preferentially cleaves ubiquitin-adducts with small leaving groups over ubiquitin–protein conjugates. Interestingly, a recent study showed that proteasome-associated UCH37 selectively removes K48-branched chains from a complex mixture of ubiquitin conjugates (see also Section 4 and Figure 2B) [14]. The topology of the K48-branching point on bifurcate chains might not be impeded by ASCL-imposed steric hindrance, but this model remains to be further explored.

### 2.3. RPN11

Unlike USP14 and UCH37, RPN11 is an ATP- and Zn^2+^-dependent deubiquitinase and also an essential subunit of the proteasome [9,10]. This DUB is positioned directly above the substrate entry pore, thus acting as a gatekeeper for substrate entry [16,41]. Remarkably, during the transition from the substrate-unbound S1 state to the substrate-engaged S3 state of the proteasome, RPN11 is shifted toward the central processing pore of the substrate translocation channel (i.e., AAA+-ATPase RPT ring) [13,26,30]. This repositioning from its offset position may provide a fundamental basis for RPN11’s activity being coupled to substrate unfolding and translocation in an ATP-dependent manner.

Another striking feature of RPN11 is the conformational switch in its Ins-1 loop near the catalytic site [42,43]. In an inactive state, the Ins-1 region forms a flexible shape that covers the catalytic groove and restricts the access of ubiquitin’s C-terminus to the active site (Figure 1C, panels (a) and (b)). In the active state or upon ubiquitinated substrate binding, the Ins-1 loop of RPN11 undergoes a conformational change to a β-hairpin structure (Figure 1C, panel (d)). This dramatic transition of the Ins-1 segment results in a stable interaction between the segment and the C-terminus of ubiquitin that allows for efficient catalysis, rather than blocking access to the active site [31,44,45]. Thus, in the process of substrate engagement and irreversible commitment, RPN11 spatially and temporally operates on the proteasome to promote protein degradation [5,13]. Interestingly, a recent cryo-EM structure of USP14-bound human proteasome reveals that the Ins-1 loop of RPN11 adopts a more extended and flexible conformation compared to those found in USP14-free S1 and S2 states (Figure 1C, panels (a)–(c)) [26]. Given the close positioning of both USP14 and RPN11 to adjacent RPTs, the two DUBs may be functionally linked through allosteric regulation of the ATPase cycle. It will be interesting to determine if the non-catalytic effect of Ubp6/USP14 is also mediated by a conformational change due to this altered Ins-1 loop configuration.

## 3. USP14: A Multi-Chain Specific and Allosteric Deubiquitinase

Ubp6/USP14 is transiently associated with the proteasome and can be easily lost during the process of proteasome isolation [8,17,19,21,22,23,46]. Still, USP14 activity can be remarkably enhanced by association with the proteasome—up to ~800-fold—suggesting its major regulatory role in proteasome function [8,28,37]. Contrary to RPN11, this enzyme operates without a requirement for ATP, and thus primarily acts on ubiquitinated substrates prior to their irreversible engagement into the degradation cycle. As observed by single encounter quench flow analysis, USP14-mediated deubiquitination occurs extremely fast (on milliseconds time scale), at least for its preferred substrates [6]. Such a rapid rate of reaction is competent for outrunning the proteasome in kinetic competition because deubiquitinated substrates will prematurely dissociate from the proteasome before the proteolytic commitment step [6,8,13]. This suggests that a unique negative feedback model, in which USP14 is activated by proteasome and then proteasomal degradation is suppressed by deubiquitination, is the basis by which USP14 serves as a critical inhibitory component of the proteasome [6,8,13].

It was once widely assumed that proteasome-bound USP14 progressively shortens the chains, one ubiquitin moiety at a time, from the substrate distal tip—the so called “distal chain trimming model” [24,47]. Although rapid chain trimming might lead to substrate release from the proteasome prior to degradation, both free and proteasome-bound Ubp6/USP14 in fact hydrolyze unanchored ubiquitin chains at an extremely slow rate, resulting in minimal cleavage even after hours of incubation [6,24,48]. This dilemma may suggest a strong substrate preference for USP14, especially in the context of the proteasome. A recent study of a complex mixture of ubiquitin–protein conjugates showed that proteasome-associated USP14 preferentially cleaves the substrates carrying multiple short and heterogeneous ubiquitin chains as fast as milliseconds to seconds (Figure 2A, left panel) [6]. The cleavage reaction also occurs on multi-monoubiquitinated substrates and more defined ubiquitinated substrates of varied length and linkage types, yet surprisingly does not occur with single chain bearing conjugates (Figure 2A, left panel) [6,7]. This striking multi-chain specific cleavage by USP14 is the first to be reported among any of ~100 DUBs, and explains why Ubp6/USP14 so poorly cleaves free chains and some isopeptide-linked ubiquitin–protein conjugates [24,48,49]. Another unexpected finding of USP14 is its en bloc cleavage mechanism (the proximal cutting at the junction of ubiquitin chains on the substrate)—a feature that it shares with RPN11 (Figure 2A, left panel) [6]. So, rather than distal chain trimming, USP14 has a very similar cleavage mechanism to RPN11, yet their influence on proteolytic outcome can be quite opposite. Recent cryo-EM-based data elucidated that USP14’s catalytic domain is positioned close to the ATPase ring of the proteasome base that is too restrained to hold a proximal ubiquitin or any folded domain (Figure 1A and Figure 2A, left panel) [6,13]. This topological constraint, which is also shared by RPN11, may drive the two enzymes to adopt the en bloc mode of activity. The key difference here, however, is that USP14 will cut the base of multi-chain conjugate until leaving the last single chain prior to irreversible commitment, so depending on the affinity or length of a remaining chain, the substrate will have a chance to be rescued (Figure 2A, left panel) [6,7,13].

Along with its catalytic impact on the substrate, Ubp6/USP14 also exerts multiple allosteric effects on the proteasome, mainly through a non-catalytic mechanism [17,18,19,32,33,50,51]. Ubp6 harboring a catalytically inactive C114A mutant strongly inhibits substrate degradation by the proteasome in vitro and in cells, which is also closely linked to deubiquitinating activity-dependent ubiquitin flux (Figure 2A, right top panel) [17,18,52]. A series of studies have demonstrated that Ubp6/USP14 enhances the ATPase activity of proteasome and opens the 20S gate upon treatment with ubiquitin, ubiquitin–protein conjugate or active site-directed probes, such as ubiquitin aldehyde (Ubal) or ubiquitin vinyl sulfone (UbVS) (Figure 2A, right bottom panel) [18,19,32,33]. These effects should be non-catalytic because the catalytically inactive C to A mutant of Ubp6/USP14 still retains this function. Moreover, the isolated UBL domain of USP14 (i.e., lacking the whole catalytic domain) and even UBLs of other assorted origins are capable of stimulating ATPase activity and peptide hydrolysis through gate opening of proteasome [50,51]. Therefore, the non-catalytic effect of Ubp6/USP14 may simply reflect the allosteric outcome of a certain conformational state of the proteasome during dynamic transition. As described above, recent cryo-EM data suggest that the Ubp6/USP14-bound proteasome strongly favors the S2 state conformation, in which the ubiquitin-charged catalytic domain of Ubp6/USP14 makes stable contacts with the base RPT ring [18,21,26]. This biased state with Ubp6/USP14 occupancy may suppress proteasome activity by preventing conformational transitions of the proteasome between the substrate-engaged and substrate-permissive states, while exhibiting the altered rate of ATP and peptide hydrolysis as collateral effects. Perhaps depending on cellular context and regulatory events, the dynamic nature of USP14 may impose a proteolysis checkpoint by sensing proteasome conformational states, and in which its catalytic activation and non-catalytic effect must be intimately tied together.

USP14 is often associated with oncogenic function, since this enzyme is overexpressed in several cancers and is positively correlated with tumor recurrence and poor prognosis [36,53,54,55,56,57]. More specifically, elevated USP14 expression has been found in various cancer types, including lung, breast and gastric cancers, pancreatic adenocarcinomas, and esophageal squamous cell carcinoma [36]. AKT-induced phosphorylation of USP14 may also promote tumor cell proliferation by regulating global proteomic turnover [27]. However, TRIM11-dependent tumor cell growth can be suppressed by USP14 overexpression in the HCT116 cell-derived xenograft model, which suggests possible opposing roles of USP14 in tumorigenesis [58]. Earlier studies of USP14 in animal physiology came from a hypomorphic ataxia (ax^J^) mutant phenotype in mice [59,60,61,62]. The reduction in USP14 caused abnormalities in structure and function at the neuromuscular junction, and the mutant showed early developmental defects in motor neurons. Interestingly, transgenic expression of ubiquitin or a catalytically inactive USP14 ameliorated the ax^J^ phenotypes in the nervous system [63,64,65,66], suggesting that the mutational defects may reflect the perturbation of both catalytic and non-catalytic functions of USP14.

Finley and colleagues have identified highly selective USP14 inhibitors, IU1 and its derivatives, by high-throughput screening and subsequent structure–activity relationship studies [8,34,37], and their specificity was also confirmed by recent co-crystal structures of inhibitor-bound USP14 [28]. When treated to the cells and in vitro, USP14 inhibitors promote the degradation of a subset of substrates by enhancing proteasome activity [8,34], indicating that these compounds may uncheck the USP14-mediated proteolytic checkpoint on the proteasome under certain conditions. Therefore, USP14 inhibitors should offer unprecedented opportunities, not only as a chemical tool to dissect the deubiquitination reaction and identify endogenous substrates, but also as a drug-like molecule to explore therapeutic potential [8,34]. Consistent with results from USP14 overexpression and loss-of-function analyses, treatment with USP14 inhibitors promotes the degradation of a variety of proteotoxic proteins within cells, including those highly implicated in neurodegenerative diseases—such as tau, TDP-43, ATXN3, and PrP^SC^ [8,34,67,68]. USP14 or IU1 sensitivity was also reported to regulate selective autophagy [69], Parkin or PINK1-mediated mitophagy [35], innate immunity [70,71], androgen receptor stability [72], metabolic syndrome [73,74], and the turnover of other endogenous substrates (see also Finley and Prado (2020) and references therein). Any governing rule for USP14’s substrate specificity remains to be further elucidated, but this DUB is likely to operate in the context of conjugate configuration and proteasome conformation in response to cellular cues.

## 4. UCH37: A Cryptic and Editing Deubiquitinase

The ubiquitin carboxyl terminal hydrolase UCH37 (aka, UCH-L5) is a proteasome RP-associated thiol protease [1]. As discussed above, UCH37 belongs to both the proteasome and INO80 complex [29,38,39,40,75,76,77,78]. Their interaction is mutually exclusive, since the ULD domain of UCH37 is the overlapping binding site for RPN13^DEUBAD^ and NFRKB^DEUBAD^ (Figure 1B). In addition, the fact that UCH37 activation in the proteasome complex is reversed in the INO80 complex may imply a unique compartmental role or functional partitioning of this enzyme. Otherwise, the exact function and substrate specificity of UCH37 remain largely elusive. This DUB has been suggested to antagonize protein degradation via editing or trimming from the distal end of polyubiquitin chains (Figure 2B, top panel) [12,47,79]. UCH37 in either free or proteasome-bound form was reported to cleave different linkage types of ubiquitin chains, despite showing much higher activity against ubiquitin adducts with small leaving groups [8,37,49,80,81]. However, UCH37 acts too slowly on isopeptide-linked ubiquitin–protein conjugates or chains, so the model proposed for USP14, whereby it regulates the dwell time of the substrate on proteasome, is hard to justify for UCH37. As noted in Section 2, the unique structural constraint of UCH37’s ASCL loop may force this DUB to hold only limited types of substrates which have the right configuration. So, UCH37 is likely to have its own peculiar substrate preference that is yet to be identified, still leaving the question of whether small ubiquitin adducts are also among its physiological substrates.

A recent study proposed that proteasome-associated UCH37 can debranch ubiquitin chains to promote substrate degradation [14]. Emerging evidence indicates that branched ubiquitin chains play essential roles in the physiological environment [82,83,84], but a branched-chain-specific DUB has so far not been discovered. Strieter’s group found that UCH37 selectively cleaves K48-linked branching points from complex mixture of chains, and its selectivity and activity can be markedly augmented in association with RPN13, at a turnover rate of 3 to 4 s [14]. This debranching activity essentially promotes the degradation of substrates that are modified with K48-linked branched chains under multi-turnover conditions in vitro and in cells (Figure 2B, bottom panel) [14]. This unexpected substrate selection strategy of UCH37 certainly demands further studies to be established.

As with USP14, UCH37 is associated with oncogenesis, although this DUB has been also implicated in many different cellular processes [13]. Elevated UCH37 level has been detected in several cancers, and its high expression is positively correlated with cancer recurrence and decreased patient survival in cervical carcinoma, esophageal squamous cell carcinoma, hepatocellular carcinoma, and epithelial ovarian cancer [85,86,87,88]. On the other hand, positive UCH37 expression was also reported as a prognostic marker for increased patient survival in colorectal cancer, pancreatic ductal adenocarcinoma, and gastric cancer [89,90,91]. At the molecular and cellular level, UCH37 is involved in a number of crucial signaling pathways for the regulation of cellular growth and fate, including TGF-β signaling, Wnt signaling, Hedgehog signaling, apoptosis, DNA replication/cell cycle and cell migration/invasion [92,93,94,95,96]. For example, UCH37’s binding to Smoothened (Smo) counteracts Smo ubiquitination and leads to the stabilization of the Smo protein and the activation of the Hedgehog signaling pathway [95]. Likewise, UCH37 was reported to deubiquitinate and subsequently stabilize Smad2/Smad3, thus stimulating TGF-β signaling in pulmonary fibrosis [92]. These findings may emphasize UCH37 as a potential therapeutic target, but the underlying mechanistic basis should be further characterized.

## 5. RPN11: An Essential and Degradation-Coupled Deubiquitinase

RPN11, which is also known as PSMD14 or POH1, is a JAMM metalloprotease class of DUB and also an integral subunit of the 26S proteasome that resides on the lid subcomplex of 19S RP [1,5]. This enzyme typically shows degradation-coupled and ATP-dependent deubiquitinating activity only in the context of the proteasome (Figure 2C) [9,10]. In isolation, RPN11 can form a hetero-dimeric complex with RPN8, which unlike free USP14, shows a highly promiscuous DUB activity [42,43]. In the proteasome complex, RPN11 is tightly placed just above the OB ring of the substrate translocation channel, implying a functional axis of energy-dependent unfolding–translocation–deubiquitination events into proteolysis [97]. As discussed earlier, RPN11 undergoes important structural changes depending on the conformational state of the proteasome. First, during the transition from latent S1 state to substrate-processing S3 state, the proteasome repositions RPN11 in a closer proximity to the substrate entry port so that the axially transverse ubiquitinated substrate can be drawn further toward RPN11’s active site for efficient catalysis [13,41,98]. Moreover, when engaged with a substrate, the Ins-1 loop within the active site displays a striking conformational switch from a flexible and loose structure (in a closed state) to a β-hairpin shape (in an active state), which allows the catalytic site to be accessible (Figure 1C) [13,26,31,42,43,44,70]. As noted above, the close positioning of RPN11 to the substrate processing pore sterically restricts cleavage in-between ubiquitin chains, thus only permitting the en bloc cleavage mode at the proximal base of chain on the substrate. Compared to USP14, however, the ATP-dependent nature of RPN11 may strictly couple this DUB to unfolding, translocation, and degradation for the committed substrates (Figure 2C).

As expected, RPN11 plays an essential role in proteasome integrity and cell viability across species [9,10,99,100,101,102,103]. The lethality of RPN11 knock-out is largely due to a defect in proteasome assembly; catalytic site mutants are viable and assembled into the intact complex, albeit severely compromised in degradation ability [9,10,99,101,103]. A number of defects caused by RPN11 loss-of-function have been reported in mammals, including c-Jun stability, double-strand DNA break response, E2F1-mediated tumor formation, and cellular reprogramming [104,105,106,107]. Recently, Deshaies and colleagues developed a potent and specific small-molecule inhibitor of RPN11, capzimin, through a series of a fragment-based drug discovery approach of metal-binding pharmacophores, high-throughput screening, and structure-activity relationship studies [108,109]. Capzimin, which is a derivative of quinoline-8-thiol, stabilized a subset of proteasome substrates in treated cells and inhibited the proliferation of cancer cells [108,109,110]. This compound mediates a distinct mode of proteasome inhibition by blocking the step of degradation-coupled deubiquitination, and indeed it exerts cytotoxicity even to the cells resistant to the proteasome inhibitor, bortezomib [108]. RPN11 inhibition may offer another exciting opportunity to target the ubiquitin–proteasome system for anti-cancer therapy.

## 6. Summary and Future Perspectives

The critical contribution of the proteasome to healthy cellular proteostasis has been increasingly recognized in recent years [111,112]. To successfully cope with various proteolytic cues or challenges, the proteasome is subject to dynamic checkpoints in the form of regulatory mechanisms, notably deubiquitination reactions. In this review, we briefly describe recent progress in understanding the roles of proteasome-associated DUBs in influencing the proteasome’s proteolytic versatility. Deubiquitination activities on the proteasome may have evolved remarkable strategies which contribute to the ability of the proteasome to adapt to the demand of selective degradation of specific substrates, while still actively processing thousands of diverse polypeptides. Future studies should address how and when DUBs selectively recognize and process various ubiquitin conjugates, what governs this selectivity, and whether each or an ensemble of the deconjugation abilities has major impacts on dynamic proteolysis. Targeting DUBs at the proteasome will also offer exciting opportunities for both restrained and induced protein degradation, and these considerations may have significant therapeutic implications in many intractable human diseases.

## Abbreviations

DUB	Deubiquitinating enzyme
RP	Regulatory particle
CP	Core particle
UBL	Ubiquitin-like
UbAMC	Ubiquitin amidomethylcoumarin
ULD	UCH37-like
ASCL	Active site crossover loop
DEUBAD	Deubiquitinase adaptor
Ubal	Ubiquitin aldehyde
UbVS	Ubiquitin vinyl sulfone
Smo	Smoothened
